# Supplementary material for: A Multi-Dataset Evaluation of Frame Censoring for Motion Correction in Task-Based fMRI
Source: Apert Neuro. Author manuscript; Available in PMC 2022 Sep 23. (PMC9506314; doi:10.52294/apertureneuro.2022.2.nxor2026)
Supplement: 1 [file NIHMS1831075-supplement-1.pdf]

Supplemental Methods

Representational similarity analysis of group-level maps. Overlap of the thresholded group-level maps ($p < 0.001$, uncorrected) was quantified using a Dice coefficient computed for all pairs of denoising approaches. The result was a 16 × 16 matrix for each dataset in which the (i,j)-th entry is the Dice coefficient quantifying overlap of the group-level map obtained using the i-th denoising strategy with that of the j-th denoising strategy. The overlap summaries were then used to explore a multivariate characterization of the denoising strategies. To this end, all pairs of the 15 dice overlap matrices were correlated and the coefficients were collected into a 15 × 15 distance matrix (distance = $1 - r$). Multidimensional scaling (MDS; Matlab function *cmdscale*) was then applied to the distance matrix to obtain a low-dimensional approximation which was plotted and examined for clustering or other patterns.

Univariate statistical analysis. Maximum t values, mean ROI effect size, and Dice test–retest values for each denoising strategy were pooled across all datasets and analyzed using a repeated-measures analysis of variance (Matlab functions *fitrm* and *rmanova*). Maximum t, effect size, and Dice test–retest score were normalized to the value obtained using no motion correction prior to pooling to account for variability across datasets. Pairwise differences were identified in post hoc testing using a Scheffe test (Matlab function *multcompare*).

An alternate evaluation of algorithm performance was explored by simply counting the number of datasets each approach gave the best result (i.e., the largest value) on a given metric. For the purpose of this analysis, FD and DVARS results were combined across all percent data loss categories, reducing the total number of categories to eight. Counts were then evaluated statistically using a binomial test (Zar 2010). Under the null hypothesis that all denoising strategies perform equally well, exhibiting the best performance on seven or more datasets by any one strategy is significant at the 0.05 level.

 CC-BY: © Jones et al.

Supplemental Results

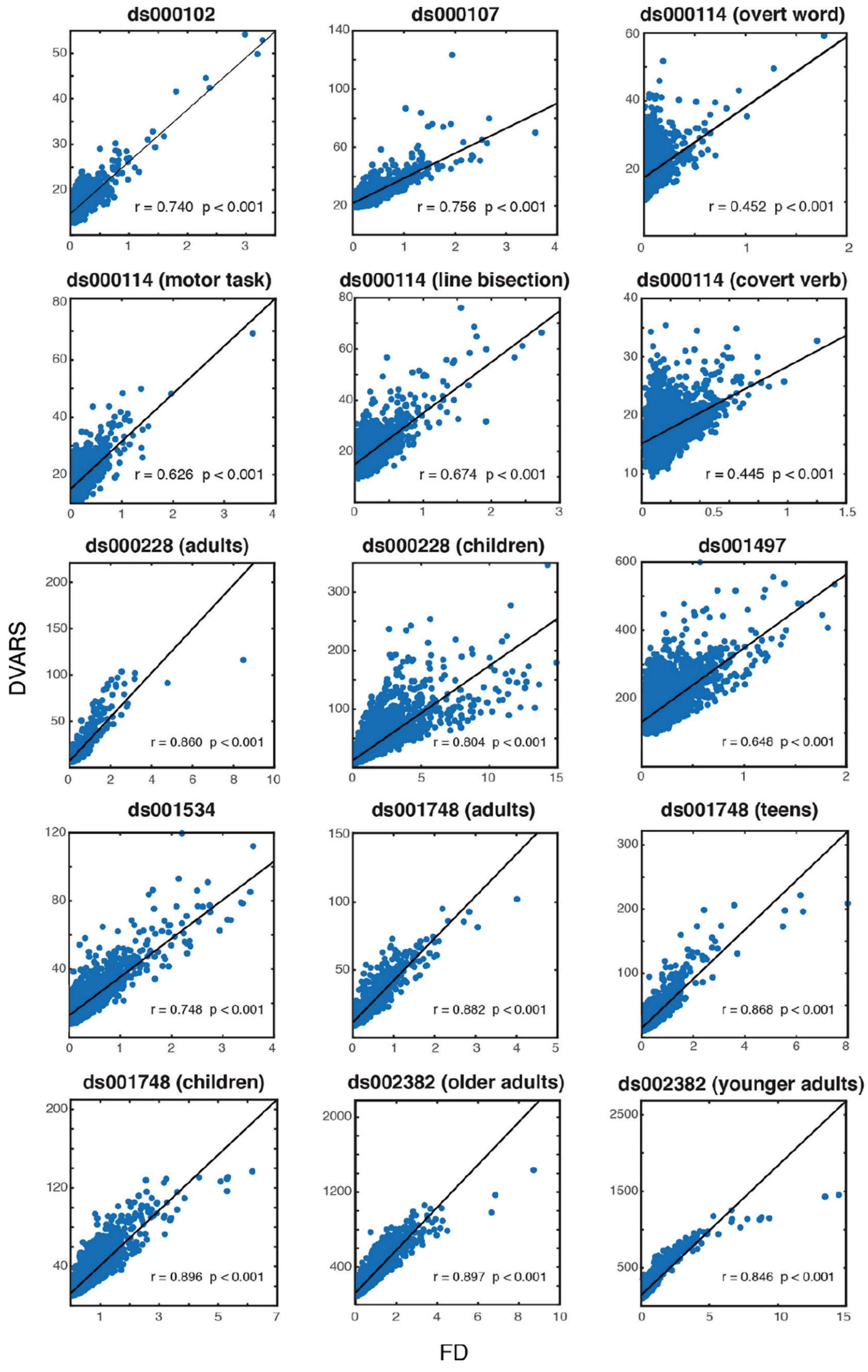

Fig. S1. Correlation of FD and DVARS for all datasets. Each point is the FD (*x*) and corresponding DVARS (*y*) for a single frame, combined across all sessions and subjects for a given dataset. Each plot includes a least-squares line fit to the data.

 CC-BY: © Jones et al.

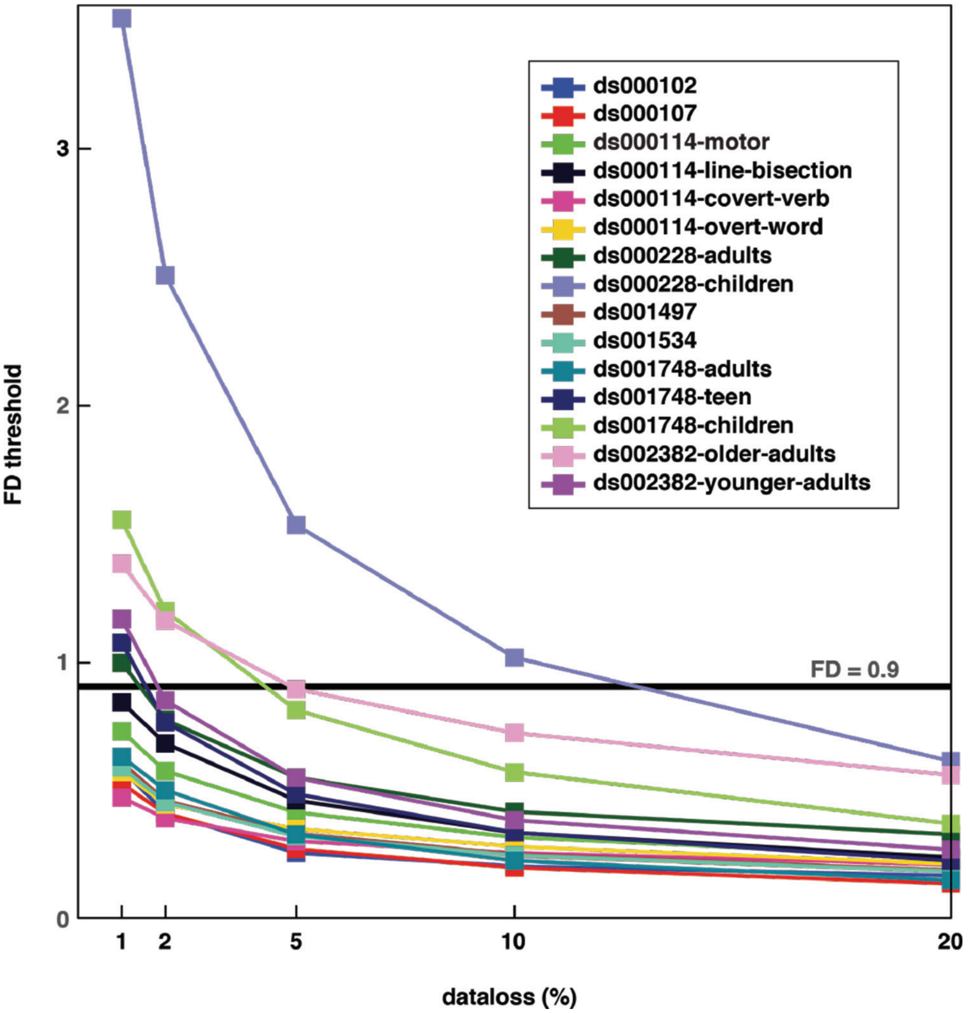

Fig. S2. FD values resulting from targeted percent data loss. The FD threshold value that resulted in 1%, 2%, 5%, 10%, or 20% data loss for a given dataset can be read from the *y*-axis. A horizontal line at FD = 0.9 is included to illustrate the data loss that would have occurred had an (arbitrary) fixed FD threshold of 0.9 been applied (given by the intersection or extrapolated intersection of the horizontal line with the graph of a dataset).

CC-BY: © Jones et al.

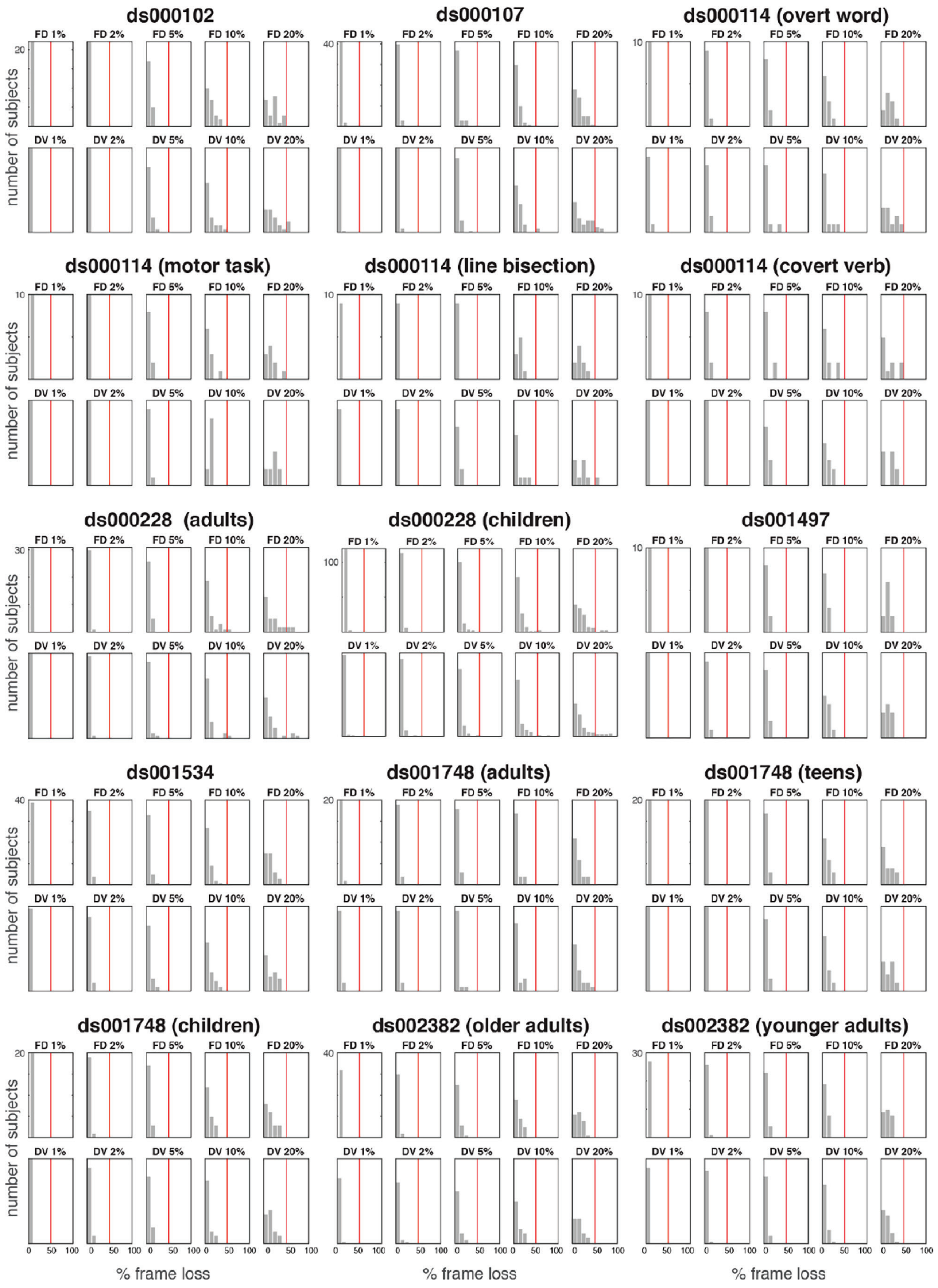

Fig. S3. **Frameloss histograms for all 15 datasets and for all FD and DVARS thresholds.** Red line indicates 50% frame loss for reference. See also Figure 2 in the main text.

 CC-BY: © Jones et al.

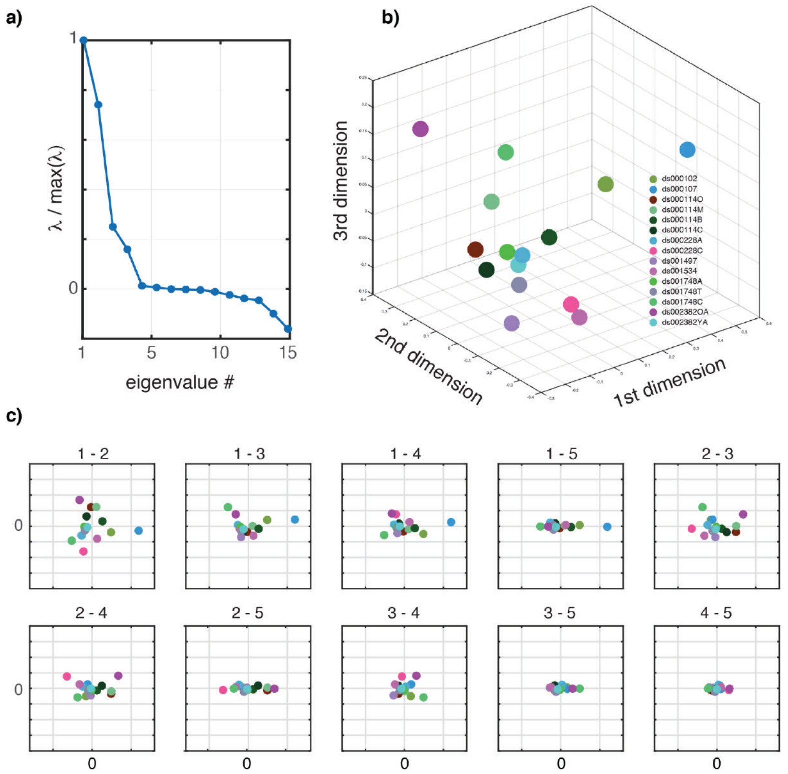

Fig. S4. Scree plot and additional MDS results. **(a)** Eigenvalue magnitude normalized to that of the largest eigenvalue returned by a multidimensional scaling of the 15 Dice overlap matrices shown in Figure 4 in the main text. Falloff suggests the dimensionality of the distance data is about five. The presence of negative eigenvalues indicates the dataspace is non-Euclidean. **(b)** Plot of first three eigen-dimensions reveals no obvious pattern in the data. **(c)** Projections of all possible pairs of the first five eigen-dimensions. The dataset ds000107 is segregated in some of the plots, but no other organization is apparent.

 CC-BY: © Jones et al.

Table S1. Some prior evaluations of motion correction in task-based fMRI

Study	# Data sets	Task(s)	*N*	Motion correction approaches	Outcome measures
Current study	8	See Table 1	405	RP6, RP24, rWLS, WDS, untrained ICA, FD- and DVAR-based frame censoring	Whole-brain and ROI maximum *t*, mean effect size, Dice
Diedrichsen and Shadmehr (2005)	1	Hand controlled cursor targeting	15	Robust weighted least squares	*t*-Score, number of suprathreshold voxels
Hoffmann et al. (2015)	1	Auditory (SPM MoAE data contaminated with clinically relevant motion)	1	Rigid body alignment in SPM, FSL, AFNI, or AIR	Count of suprathreshold voxels, specious identification of motion
Huang et al. (2008)	1	Reading aloud	13	Linear interpolation over contaminated volumes	Significant voxel counts
Johnstone et al. (2006)	1	Go/no-go, N-back	33	Rigid body registration with versus without including six realignment parameters as covariates	Whole-brain maximum *t* and cluster extent
Kay et al. (2013)	11**	Visual	1–3	Custom (GLMDenoise), ICA	Cross validation
Kochiyama et al. (2005)	1	Finger tapping	4	ICA	ROC true versus false-positive fraction
Lemmin et al. (2010)	1	Arm motion	7	Model-based motion estimation, RP6, rWLS	Reduction in ventricle activation
Liao et al. (2006)	1	Motor	10	Custom ICA	Standard deviation reduction in activated voxels
Mayer et al. (2019)	2*	AX continuous performance task, multimodal attention	110	RP12, RP24, untrained ICA, trained ICA	Percent change of true and false activation
Middlebrooks et al. (2017)	1	Motor task, language task	12	Realignment, DVARS scrubbing, trained ICA, ICA+scrubbing	Increased *z*-scores in areas of expected activation
Oakes et al. (2005)	1	Go/No-go, N-back	40	Rigid body registration in AFNI, AIR, BrainVoyager, FSL, or SPM	Maximum *t* and cluster extent
Siegel et al. (2014)	4*	String matching, rule switching, posner task	88	Motion estimates as nuisance regressors versus FD-thresholded frame censoring	Change in β estimate, error variance, or *t*-score
Tierney et al. (2016)	1	Sentence comprehension and generation	42	Custom algorithm (FIACH) compared with RP6, RP24, FD frame censoring, rWLS, tCompCorr	ROI restricted *t*-values and cluster extent
Tohka et al. (2008)	2***	Tone counting, weather prediction	32	Trained ICA	Change in Z-scores
Wilke and Baldeweg (2019)	3	Verb generation, hand motor task, language task	84	Custom algorithm	*t*-Value, # activated voxels, SNR
Xu et al. (2014)	1	Overt speech	18	Custom ICA	PET cross validation

* Same scanner, multiple cohorts

** 11 task variations run on a single cohort

*** A training and a test cohort on the same scanner/tasks

Table S2. Acquisition details for datasets analyzed

Dataset	Reference	Scanner	Field strength (T)	TR (s)	Voxel size (mm)
ds000102	Kelly et al. (2008)	Siemens Allegra	3	2	3 × 3 × 4
ds000107	Duncan et al. (2009)	Siemens Avanto	1.5	3	3 × 3 × 3
ds000114	Gorgolewski et al. (2013)	GE Signa HDxt	1.5	2.5	4 × 4 × 4
ds000228	Richardson et al. (2018)	Siemens Tim Trio	3	2	3 × 3 × 3
ds001497	Lewis-Peacock and Postle (2008)	GE Signa VH/I	3	2	3.75 × 3.75 × 4
ds001534	Courtney et al. (2018)	Philips Intera Achieva	2.5	2.5	3 × 3 × 3
ds001748	Fynes-Clinton et al. (2019)	Siemens Magnetom Trio	3	3	2.5 × 2.5 × 2.5
ds002382	Rogers et al. (2020)	Siemens Prisma	3	3.07	2 × 2 × 2

 CC-BY: © Jones et al.

Table S3. DVARS and FD values at percent frameloss

	FD					DVARS				
Dataset	1%	2%	5%	10%	20%	1%	2%	5%	10%	20%
ds000102	22.1	20.2	18.5	17.5	16.9	0.58	0.41	0.25	0.20	0.16
ds000107	32.3	29.5	26.8	25.3	24.2	0.52	0.41	0.27	0.20	0.13
ds000114 bisection	35.4	30.8	25.9	23.1	20.8	0.84	0.68	0.46	0.33	0.24
ds000114 covert verb	25.5	23.8	22.1	20.6	19.0	0.47	0.39	0.30	0.25	0.21
ds000114 lips motor	31.1	28.1	24.3	22.1	20.1	0.73	0.57	0.41	0.31	0.24
ds000114 overt word	33.7	31.8	28.6	26.3	23.7	0.57	0.44	0.35	0.28	0.21
ds000228 adults	30.1	25.3	20.9	17.4	15.1	0.99	0.77	0.55	0.41	0.33
ds000228 children	72.3	59.1	42.1	31.6	23.5	3.50	2.50	1.53	1.01	0.61
ds001497	284.6	251.3	219.4	198.3	179.6	0.60	0.45	0.33	0.24	0.18
ds001534	28.2	24.1	20.7	18.8	17.1	0.59	0.45	0.32	0.24	0.18
ds001748 adults	31.0	27.4	22.0	19.2	16.4	0.63	0.50	0.32	0.22	0.15
ds001748 children	58.0	50.5	38.4	30.0	23.5	1.55	1.19	0.81	0.57	0.37
ds001748 teen	58.7	50.1	35.9	28.6	23.7	1.07	0.76	0.48	0.33	0.22
ds002382 older adult	463.4	410.1	343.6	300.8	258.0	1.38	1.16	0.89	0.72	0.56
ds002382 young adult	382.0	324.3	258.8	222.1	193.2	1.16	0.85	0.55	0.38	0.27

Additional Dataset Details

Details of datasets used in the study are summarized below, including challenges or irregularities we encountered during analysis.

Accession Number: ds000102

Publication: Kelly et al. (2008)

Task: Slow event-related Eriksen flanker

Task details: Participants used one of two buttons to indicate the direction of a central arrow in an array of five arrows. In congruent trials, the flanking arrows pointed in the same direction as the central arrow; in more demanding incongruent trials, the flanking arrows pointed in the opposite direction.

Acquisition: Siemens Allegra 3.0 T (TR = 2000 ms; TE = 30 ms; flip angle = 80, 40 slices, matrix = 64 × 64; FOV = 192 mm; acquisition voxel size = 3 × 3 × 4 mm).

Number of subjects: 26; Age: 22–50 years (mean 32)

Contrast evaluated: incongruent correct > congruent correct

ROI: Neuroquery search term "incongruent task" and thresholded $Z > 3$

Notes: Functional data include a pronounced periodic artifact that appears unrelated to motion.

 CC-BY: © Jones et al.

Accession Number: ds000107

Publication: Duncan et al. (2009)

Task: One-back

Task details: A one-back task was used with four categories of visual stimuli: written words, pictures of common objects, scrambled pictures of the same objects, and consonant letter strings. Subjects were instructed to press a button if the stimulus was identical to the preceding stimulus (12.5% of the stimuli were targets). Each block consisted of 16 trials from a single category presented one every second. A trial began with a 650 ms fixation cross, followed by the stimulus for 350 ms.

Acquisition: Siemens Avanto 1.5 T. The functional data were acquired with a gradient-echo EPI sequence (TR = 3000 ms; TE = 50 ms; FOV = 192 × 192; matrix = 64 × 64, voxel size = 3 × 3 × 3 mm).

Number of subjects: 45 (23 Male); Age: 19–38 years (mean 25)

Contrast evaluated: Words > 0

ROI: 5 mm sphere at [−42 −62 −16] (left ventral occipital–temporal cortex). Coordinates are from Table 2 of Duncan et al. (2009).

Notes: Data from six subjects were excluded: two because of corrupt data, three due to data modeling errors that could not be corrected, and one due to an incompatible contrast definition. Removed spaces from event names in BIDS .tsv files.

Accession Number: ds000114

Publication: Gorgolewski et al. (2013)

Tasks: (i) lip movement, (ii) covert verb generation, (iii) overt word generation, (iv) line bisection. All tasks used the same subjects.

Task details: Lip movement: Lip poaching (15 s) interleaved with fixation at a cross (15 s). Covert verb generation: Subjects instructed to think of a verb following presentation of a random noun for 1 s. Overt word generation: Repeat words aloud presented via headphones; 30 s task / 30 s rest repeated six times. Line bisection: Judge by button press if a horizontal line was bisected exactly in the middle (landmark) or if a horizontal line was crossed or not crossed (detection). Randomized presentation of six correct and four incorrect lines (525 ms presentation / 1100 ms response) in eight blocks.

Acquisition: GE Signa HDxt 1.5 T scanner with an 8-channel phased-array head coil. FOV = 256 × 256 mm, voxel size = 4 × 4 × 4 mm, slice thickness 4 mm, 30 slices per volume, interleaved slices order, acquisition matrix 64 × 64, flip angle = 90, TE = 50 ms, TR = 2.5 s, except for overt word repetition in which sparse sampling was used (TR = 5 s, "real TR"—which we assumed meant "acquisition time/TA" = 2.5 s). Subjects were scanned twice, either two or three days apart.

Number of subjects: 10 (4 Male); age: 50–58 years (median 52.5).

Contrast evaluated: (i) Motor task: lip > hand+foot, (ii) Covert verb: task > 0, (iii) Overt word: task > 0, (iv) Line bisection: landmark > detection

ROI: (i) 5 mm spheres in bilateral motor cortex ([−56, −6, 26] and [62, 0, 28]). Coordinates taken from (Pulvermüller et al. 2006), (ii) Covert verb: Broca's Area (BA 44 + BA 45 (left hemisphere only) from Anatomy Toolbox), (iii) Overt Word: same as lip motor task, (iv) Line bisection: lateral visual cortex (Anatomy Toolbox hoc3v+doc4v+hoc4lp).

Notes: The motor data included finger and foot tapping tasks that were not used because a mixture of left- and right-handed activation precluded straightforward second-level modeling. Subject 10 was excluded from the line bisection analysis as first level maps suggest the subject misunderstood task instructions (activation is left/right reversed).

Accession Number: ds000228

Publication: Richardson et al. (2018)

Task: Film viewing

Task details: Subjects viewed a 5.6-minute animated film with scenes classified as presenting either "pain" or "theory of mind" events.

 CC-BY: © Jones et al.

Acquisition: 3T Siemens Tim Trio using a standard Siemens 32-channel head coil. T1-weighted structural images were collected in 176 interleaved sagittal slices with 1 mm isotropic voxels (GRAPPA parallel imaging, acceleration factor of 3; adult coil: FOV: 256 mm; kid coils: FOV: 192 mm). Functional data were collected with a gradient-echo EPI sequence in 32 interleaved near-axial slices aligned with the anterior/posterior commissure and covering the whole brain (EPI factor: 64; TR: 2 s, TE: 30 ms, flip angle: 90). Voxel size: Adults 3.13 × 3.13 × 3.13 mm; children either 3 × 3 × 3 mm or 3.13 × 3.13 × 3.13 mm.

Number of subjects: Adults: 33 (20 female); age: 18–39 years (mean: 24.8). Children: 123 (64 female); age: 3.5–12 years; mean: 6.7).

Contrast evaluated: pain > theory of mind

ROI: Regions listed in Supplementary Table 2 of Richardson et al. (2018), modeled as a collection of 5 mm spheres.

Notes: There were insufficient sessions in this data for test−retest evaluation. Event files were missing from the original OpenNeuro listing and were added manually using information provided in the description. The event timing provided was converted to seconds from scans using the TR information.

Accession Number: ds001497

Publication: Lewis-Peacock and Postle (2008)

Task: Stimulus judgment / memory

Task details: Subjects viewed a total of 90 stimuli drawn from three categories: 30 famous people, 30 famous locations, and 30 common objects. They indicated (on a four-point Likert scale, using a stimulus–response box) how much they liked the celebrity, how much they would like to visit the location, or how often they encountered the object in everyday life.

Acquisition: GE Signa VH/I 3T scanner. T1 (30 axial slices, 0.9375 × 0.9375 × 4 mm). Functional images: gradient-echo echo-planar (TR = 2000 ms; TE = 50 ms; 64 × 64 matrix coplanar with the T1 acquisition, voxel size = 3.75 × 3.75 × 4 mm).

Number of subjects: 10 (7 male); age: 19–32 years.

Contrast evaluated: Face > 0

ROI: Bilateral fusiform face area (Neuroquery search term "FFA" and thresholded Z > 3)

Notes: There were a total of six sessions in the data which were split into even and odd sessions for test–retest evaluation. The data on OpenNeuro is only the "LTM" portion of the experiment. Data from a working memory task described in the associated publication are not included.

Accession Number: ds001534

Publication: Courtney et al. (2018)

Task: Food images paired with textural calorie content

Task details: Participants first viewed images of food paired with an accompanying image number ("foodimage"), and subsequently viewed these same food images paired with the corresponding calorie information ("calorieimage"). The presentation sequence of food images and jittered fixation trials were pseudo-randomized.

Acquisition: Philips Intera Achieva scanner. Anatomical images were acquired using gradient-echo sequence (TR = 9.9 ms; TE = 4.6 ms; flip angle = 8; 1 × 1 × 1 mm voxels). Functional images were collected using T2* fast field echo (TR = 2.5 seconds, TE = 35 ms, flip angle = 90, voxel size = 3 × 3 × 3 mm).

Number of subjects: 50 (50 M); age: 18–22 years (mean 19.7).

Contrast evaluated: labeled image > not labeled

ROI: Bilateral inferior parietal cortex (Anatomy Toolbox IPC_PF + IPC_PFcm + IPC_PFm + IPC_PFop + IPC_PFt + PIC_PGa + IPC + PGp).

Notes: Functional images in this data were scaled by 0.02 prior to processing.

 CC-BY: © Jones et al.

Accession Number: ds001748

Publication: Fynes-Clinton et al. (2019)

Task: Memory retrieval including autobiographical, episodic, or semantic conditions

Task details: One of 25 images of everyday life events was presented for 4 s, followed by a retrieval cue screen for 8 seconds during which participants retrieve different long-term memories. The type of memory retrieval was manipulated by adjusting the response screen to cue the retrieval of either personal experience (AM), general knowledge and factual information (SM), or questions about the content of the cue images (EM).

Acquisition: 3T Siemens scanner equipped with a 32-channel head coil. Structural: 176 slices sagittal; 1 mm isotropic volume; TR = 4000 ms; TE = 2.89 ms; FOV = 256 mm. Functional: T2*-weighted echo-planar image pulse sequence (45 slices, 2.5 mm slice thickness; voxel size = 2.5 × 2.5 × 2.5 mm, TR = 3000 ms; TE = 30 ms; FOV = 190 mm; flip angle = 90).

Number of subjects: 62 (32M); age: 10–35 years (see Notes).

Contrast evaluated: task > control

ROI: Inferior frontal gyrus (Neuroquery search term "IFG" thresholded Z > 3)

Notes: Data comprised three cohorts: children (10–12; *n* = 21), adolescents (14–16; *n* = 20), and young adults (20–35; *n* = 22) that were analyzed separately. There were insufficient data for test–retest evaluation. The tsv file for child-20 contains a typo with "semantic" mislabeled as "semanti" and autobio.tsv is empty for child-13. These subjects were excluded.

Accession Number: ds002382

Publication: Rogers et al. (2020)

Task: Speech comprehension in noise

Task details: Subjects were presented auditory stimuli via MR-compatible headphones consisting of words (monosyllabic consonant–vowel–consonant), silence, and noise (single-channel noise vocoded words) in two sessions of passive listening and two sessions of word repetition in which participants were asked to repeat aloud the presented word. Responses in the repeat condition were recorded and scored as either correct or incorrect.

Acquisition: MRI data were acquired using a Siemens Prisma scanner (Siemens Medical Systems) at 3 T equipped with a 32-channel head coil. Scan sequences began with a T1-weighted structural volume using an MPRAGE sequence (TR = 2.4 s, TE = 2.2 ms, flip angle = 8, 300 × 320 matrix, voxel size = 0.8 mm isotropic). Functional images were acquired using a multiband echo-planar imaging sequence (TR = 3.07 s, TA = 0.770 s, TE = 37 ms, flip angle = 37, voxel size = 2 × 2 × 2 mm, multiband factor = 8).

Number of subjects: Young adults: *n* = 29 (19 female); age: 19–30 years (mean = 23.8). Older adults: *n* = 32 (17 female); age 65–81 years (mean = 71.0).

Contrast evaluated: repeat word > noise

ROI: 5 mm spheres in bilateral motor cortex ([−56, −6, 26] and [62, 0, 28]). Coordinates taken from Pulvermüller et al. (2006)

Notes: Young adults and older adults were analyzed separately.

 CC-BY: © Jones et al.

REFERENCES

1. Courtney AL, PeConga EK, Wagner DD, Rapuano KM. Calorie information and dieting status modulate reward and control activation during the evaluation of food images. PLoS One. 2018 Nov 2;13(11):e0204744.
2. Diedrichsen J, Shadmehr R. Detecting and adjusting for artifacts in fMRI time series data. Neuroimage. 2005 Sep;27(3):624–34.
3. Duncan KJ, Pattamadilok C, Knierim I, Devlin JT. Consistency and variability in functional localisers. Neuroimage. 2009;46(4):1018–26.
4. Fynes-Clinton S, Marstaller L, Burianová H. Differentiation of functional networks during long-term memory retrieval in children and adolescents. Neuroimage. 2019 May 1;191:93–103.
5. Gorgolewski KJ, Storkey A, Bastin ME, Whittle IR, Wardlaw JM, Pernet CR. A test-retest functional MRI dataset for motor, language and spatial attention functions [Internet]. GigaScience Database; 2013. Available from: http://gigadb.org/dataset/100051
6. Hoffmann M, Carpenter TA, Williams GB, Sawiak SJ. A survey of patient motion in disorders of consciousness and optimization of its retrospective correction. Magn Reson Imaging. 2015 Apr;33(3):346–50.
7. Huang J, Francis AP, Carr TH. Studying overt word reading and speech production with event-related fMRI: a method for detecting, assessing, and correcting articulation-induced signal changes and for measuring onset time and duration of articulation. Brain Lang. 2008 Jan;104(1):10–23.
8. Johnstone T, Ores Walsh KS, Greischar LL, Alexander AL, Fox AS, Davidson RJ, et al. Motion correction and the use of motion covariates in multiple-subject fMRI analysis. Hum Brain Mapp. 2006 Oct;27(10):779–88.
9. Kay KN, Rokem A, Winawer J, Dougherty RF, Wandell BA. GLMdenoise: a fast, automated technique for denoising task-based fMRI data. Front Neurosci. 2013 Dec 17;7:247.
10. Kelly AMC, Uddin LQ, Biswal BB, Castellanos FX, Milham MP. Competition between functional brain networks mediates behavioral variability. Neuroimage. 2008 Jan 1;39(1):527–37.
11. Kochiyama T, Morita T, Okada T, Yonekura Y, Matsumura M, Sadato N. Removing the effects of task-related motion using independent-component analysis. Neuroimage. 2005 Apr 15;25(3):802–14.
12. Lemmin T, Ganesh G, Gassert R, Burdet E, Kawato M, Haruno M. Model-based attenuation of movement artifacts in fMRI. J Neurosci Methods. 2010 Sep 30;192(1):58–69.
13. Lewis-Peacock JA, Postle BR. Temporary activation of long-term memory supports working memory. J Neurosci. 2008 Aug 27;28(35):8765–71.
14. Liao R, McKeown MJ, Krolik JL. Isolation and minimization of head motion-induced signal variations in fMRI data using independent component analysis. Magn Reson Med. 2006 Jun;55(6):1396–413.
15. Mayer AR, Ling JM, Dodd AB, Shaff NA, Wertz CJ, Hanlon FM. A comparison of denoising pipelines in high temporal resolution task-based functional magnetic resonance imaging data. Hum Brain Mapp. 2019 Sep;40(13):3843–59.
16. Middlebrooks EH, Frost CJ, Tuna IS, Schmalfuss IM, Rahman M, Old Crow A. Reduction of motion artifacts and noise using independent component analysis in task-based functional MRI for preoperative planning in patients with brain tumor. AJNR Am J Neuroradiol. 2017 Feb;38(2):336–42.
17. Oakes TR, Johnstone T, Ores Walsh KS, Greischar LL, Alexander AL, Fox AS, et al. Comparison of fMRI motion correction software tools. Neuroimage. 2005 Nov 15;28(3):529–43.
18. Pulvermüller F, Huss M, Kherif F, Moscoso del Prado Martin F, Hauk O, Shtyrov Y. Motor cortex maps articulatory features of speech sounds. Proc Natl Acad Sci U S A. 2006 May 16;103(20):7865–70.
19. Richardson H, Lisandrelli G, Riobueno-Naylor A, Saxe R. Development of the social brain from age three to twelve years. Nat Commun. 2018 Mar 12;9(1):1027.
20. Rogers CS, Jones MS, McConkey S, Spehar B, Van Engen KJ, Sommers MS, et al. Age-related differences in auditory cortex activity during spoken word recognition. Neurobiol Lang. 2020 Oct;1(4):452–73.
21. Siegel JS, Power JD, Dubis JW, Vogel AC, Church JA, Schlaggar BL, et al. Statistical improvements in functional magnetic resonance imaging analyses produced by censoring high-motion data points. Hum Brain Mapp. 2014;35:1981–96.
22. Tierney TM, Weiss-Croft LJ, Centeno M, Shamshiri EA, Perani S, Baldeweg T, et al. FIACH: a biophysical model for automatic retrospective noise control in fMRI. Neuroimage. 2016 Jan 1;124(Pt A):1009–20.
23. Tohka J, Foerde K, Aron AR, Tom SM, Toga AW, Poldrack RA. Automatic independent component labeling for artifact removal in fMRI. Neuroimage. 2008 Feb 1;39(3):1227–45.
24. Wilke M, Baldeweg T. A multidimensional artefact-reduction approach to increase robustness of first-level fMRI analyses: censoring vs. interpolating. J Neurosci Methods. 2019 Apr 15;318:56–68.
25. Xu Y, Tong Y, Liu S, Chow HM, AbdulSabur NY, Mattay GS, et al. Denoising the speaking brain: toward a robust technique for correcting artifact-contaminated fMRI data under severe motion. Neuroimage. 2014 Dec;103:33–47.
26. Zar JH. Biostatistical Analysis. 5th ed. Prentice-Hall/Pearson. 2010.

 CC-BY: © Jones et al.
